# Fine Mapping of Two Interacting Loci for Transmission Ratio Distortion in Rice (*Oryza sativa* L.)

**DOI:** 10.3389/fpls.2022.866276

**Published:** 2022-03-29

**Authors:** Chaopu Zhang, Jilin Wang, Xiongfeng Xiao, Dianwen Wang, Zhiyang Yuan, Xiaodan Zhang, Wenqiang Sun, Sibin Yu

**Affiliations:** ^1^National Key Laboratory of Crop Genetic Improvement, Huazhong Agricultural University, Wuhan, China; ^2^College of Plant Science and Technology, Huazhong Agricultural University, Wuhan, China; ^3^Hubei Hongshan Laboratory, Huazhong Agricultural University, Wuhan, China

**Keywords:** rice, reproductive isolation, transmission ratio distortion, allele frequency, gametic selection, epistatic interaction

## Abstract

Transmission ratio distortion (TRD) denotes the observed allelic or genotypic frequency deviation from the expected Mendelian segregation ratios in the offspring of a heterozygote. TRD can severely hamper gene flow between and within rice species. Here, we report the fine mapping and characterization of two loci (*TRD4.1* and *TRD4.2*) for TRD using large F_2_ segregating populations, which are derived from rice chromosome segment substitution lines, each containing a particular genomic segment introduced from the *japonica* cultivar Nipponbare (NIP) into the *indica* cultivar Zhenshan (ZS97). The two loci exhibited a preferential transmission of ZS97 alleles in the derived progeny. Reciprocal crossing experiments using near-isogenic lines harboring three different alleles at *TRD4.1* suggest that the gene causes male gametic selection. Moreover, the transmission bias of *TRD4.2* was diminished in heterozygotes when they carried homozygous *TRD4.1*^ZS97^. This indicates an epistatic interaction between these two loci. *TRD4.2* was mapped into a 35-kb region encompassing one candidate gene that is specifically expressed in the reproductive organs in rice. These findings broaden the understanding of the genetic mechanisms of TRD and offer an approach to overcome the barrier of gene flow between the subspecies in rice, thus facilitating rice improvement by introgression breeding.

## Introduction

Reproductive isolation is regarded as a driving force in the process of evolution in various species (Ouyang and Zhang, 2013; Baack et al., 2015). The development of reproductive isolation relies on the accumulation of genic incompatibilities, which can lead to non-Mendelian inheritance of alleles and genotypes in the offspring of heterozygotes (or hybrids) (Fishman et al., 2008; Leppälä et al., 2013). Transmission ratio distortion (TRD) is a naturally occurring phenomenon in which one allele is preferentially transmitted to the progeny than the opposite allele in hybrids between species (Jenczewski et al., 1997; Casellas et al., 2012). If one allele at a locus can reduce gametic or zygotic fitness, then the genomic regions linked to it will cause distorted allele or genotype frequencies in heterozygotes (Vogl and Xu, 2000; Xu et al., 2013). TRD is frequently observed and characterized in intraspecific or interspecific segregating populations from various plant species, such as *Arabidopsis* (Leppälä et al., 2013; Seymour et al., 2019), cotton (Chandnani et al., 2017; Dai et al., 2017), wheat (Kumar et al., 2007), and rice (Koide et al., 2008a,b, 2012; Li et al., 2017, 2019). In particular, TRD is one of the primary origins of reproductive isolation or speciation and can severely hamper the exchange of genes among rice subspecies. Understanding the mechanisms responsible for TRD is important for using agriculturally interesting alleles from rice germplasm.

Transmission ratio distortion occurs before or after fertilization due to various reasons, such as meiotic drive, gametic competition, inbreeding depression, and hybrid incompatibilities (Huang et al., 2013; Fishman and McIntosh, 2019). It can be caused either by incompatible allelic interaction at a single-locus (Bauer et al., 2007; Corbett-Detig et al., 2013) or by two or multi-loci interaction, in which one gene effect is dependent on the presence/absence of other genes (Giesbers et al., 2019). With regard to rice, numerous loci/regions have been identified for TRD (Li et al., 2017, 2019; Zhang et al., 2020a). A few of them have been cloned and characterized in rice. They revealed that a given locus usually consists of multiple tightly linked genes and it is easily affected by the complex genetic background. For example, several well-known killer–protector and toxin–antidote segregation distortion systems that consist of multiple tightly linked genes have been identified. A killer–protector system contains three closely linked genes at the *S5* locus that regulate both hybrid fertility and segregation distortion (Yang et al., 2012). The toxin–antidote system of *qHMS7* contains two tightly linked genes (*ORF2* and *ORF3*), of which *ORF2* encodes a toxic genetic element that can kill the pollen without the protection of *ORF3*, leading to segregation distortion in heterozygotes (Yu et al., 2018). The *S1* locus, constituted of three genes *S1A4, S1TPR*, and *S1A6*, also demonstrates a killer–protector system that can eliminate the gametes carrying the Asian allele (*S1-s*), resulting in a preferential transmission of the African rice *S1* allele to the progeny (Xie et al., 2017, 2019). TRD might occur due to two or multi-loci interactions, such as S27/S28, DPL1/DPL2, and S25/S24 regions (Mizuta et al., 2010; Yamagata et al., 2010; Kubo et al., 2011; Nguyen et al., 2017). Despite these well-investigated examples, understanding the genetic and molecular mechanisms of TRD is still incomplete. Therefore, it is critical to identify more loci and candidate genes for dissecting the genetic and molecular basis of such a complex phenomenon.

Our previous studies, using backcross inbred lines (BIL) derived from an intersubspecific cross of the *japonica* cultivar Nipponbare (NIP) and the *indica* cultivar 9,311 identified and validated a number of genomic regions for TRD in rice (Zhang et al., 2020a). Two (*TRD4.1* and *TRD4.2*) were identified on chromosome 4 in which alleles were significantly skewed toward 9,311 in the BIL. In this study, the main objective is to fine-map these two TRD loci and identify their interaction pattern using several F_2_ segregating populations, which were derived from chromosome segment substitution lines, each containing a particular NIP genomic segment with the Zhenshan 97 (ZS97) background in rice. We also used a reciprocal crossing approach to detect whether *TRD4.1* is involved in male or female gametic selection. Our findings will help shed light on the genetic mechanisms underlying TRD to improve the utilization of subspecific introgression breeding in rice.

## Materials and Methods

### Plant Materials

A set of 148 chromosome segment substitution lines (CSSLs) was derived from the inter-subspecific cross between two genome-sequenced rice cultivars, *japonica* Nipponbare (NIP, as the donor) and *indica* Zhenshan97 (ZS97, as the recurrent parent), using a backcross scheme with a marker-assisted selection approach (Sun et al., 2015; Zhang et al., 2020b). A CSSL that harbors two loci/regions of interest was selected to cross with ZS97 to develop an F_1_ hybrid. Deviation of the allele and genotype frequencies from Mendelian expectations at a target locus was assessed in segregating the progeny of the hybrid. Furthermore, from the CSSL-derived progenies, several independent near-isognice lines (NILs), each containing only a single introduced heterozygous segment covering either *TRD4.1* or *TRD4.2* in the otherwise uniform background of ZS97, were developed. Six independent segregating populations were derived from relevant NILs to validate TRD loci. To delimit *TRD4.1* and *TRD4.2*, two large segregated populations were generated to select recombinant individuals per locus. The recombinants with heterozygous target regions were self-crossed to generate progeny for phenotyping TRD. In addition, the reported BIL population from the cross of NIP and 9,311, which were genotyped by the genotyping-by-sequencing method (Yuan et al., 2019), was also used for the analysis of epistatic interaction of particular TRD loci.

For reciprocal cross to test gametic selection, a line (named as NZ) carrying *TRD4.1* at a heterozygous state was first obtained by crossing ZS97 with a NIL that harbors the NIP alleles at *TRD4.1*. Then, two reciprocal populations were generated by crossing NZ as male or female parent with a developed NIL (named as MM) that contains introduced Minghui 63 (MH) alleles at *TRD4.1* (Chen et al., 2018). Both NZ and MM have the same genetic background as ZS97.

Uniformly germinated seeds were planted on 96-well plates with the bottoms removed (Li et al., 2015), and the plates were placed in a growth chamber (Dongnan, Ningbo, China) or a greenhouse at 30°C under 16-h light/8-h dark conditions. The 7-day seedlings were used for genotype analysis. Some lines and segregating populations were grown in the experimental field of Huazhong Agricultural University (HAU) in Wuhan (30.48N, 114.2E), China. Each line was planted in a row with 10 individual spacings of 16.7 cm × 26.6 cm for genotyping. The field management was managed according to the local standard practices.

### DNA Extraction and Genotype Analysis

Genomic DNA was extracted from young seedling leaves, as described previously (Zhang et al., 2020b). The genome-wide genotyping of CSSLs was conducted using the RICE 6K array (Sun et al., 2015; Chen et al., 2018). For the genotyping of segregating populations, a number of polymorphic markers, including simple sequence repeat and insertion/deletion markers, were designed (Supplementary Table 1) and used for PCR reaction following the described procedure (Panaud et al., 1996).

### Transmission Ratio Distortion Analysis

The allele and genotype frequencies were assayed by several polymorphic markers in a segregated population. The statistical Chi-square test (χ^2^) was performed using *chisq.test* function of R^1^ to determine whether the observed frequencies are distorted from Mendelian segregation ratios in any segregated population. A genomic region with two or more distorted consecutive markers was considered as one with TRD. Also, the TRD analysis in two subsets of the BIL population was performed using a single-marker analysis model in QTL IciMapping (v4.1), as described previously (Zhang et al., 2020a). A significance level of LOD > 5.0 was set as the threshold to declare the presence of a putative TRD effect in a given bin/marker.

### Gametic and Zygotic Selection

To determine the cause of TRD (e.g., gametic or zygotic selection) at a given locus, the allele and genotype (NIP and ZS97) frequencies were assayed to test if they fit the Mendelian ratios, respectively. The comparative patterns of the allele and genotype frequencies may infer gametic or zygotic selection in multiple F_2_ populations. If the allele frequency does fit the theoretical ratio of 1:1, and the genotype frequency is biased to 1: 2: 1, the TRD may be raised by zygotic factors. In this regard, an additional test is also performed to determine whether the observed heterozygote frequency deviated from the theoretical genotype frequency (0.5) in the F_2_ populations (Fishman and McIntosh, 2019). If the allele frequency is distorted from 1:1, and the genotype frequency fits the ratio of 1: 2: 1, the TRD may result from gametic factors. If there is a distortion of both allele and genotype frequencies from the Mendelian ratios, then the TRD is due to both gametic and zygotic factors.

**FIGURE 1:**
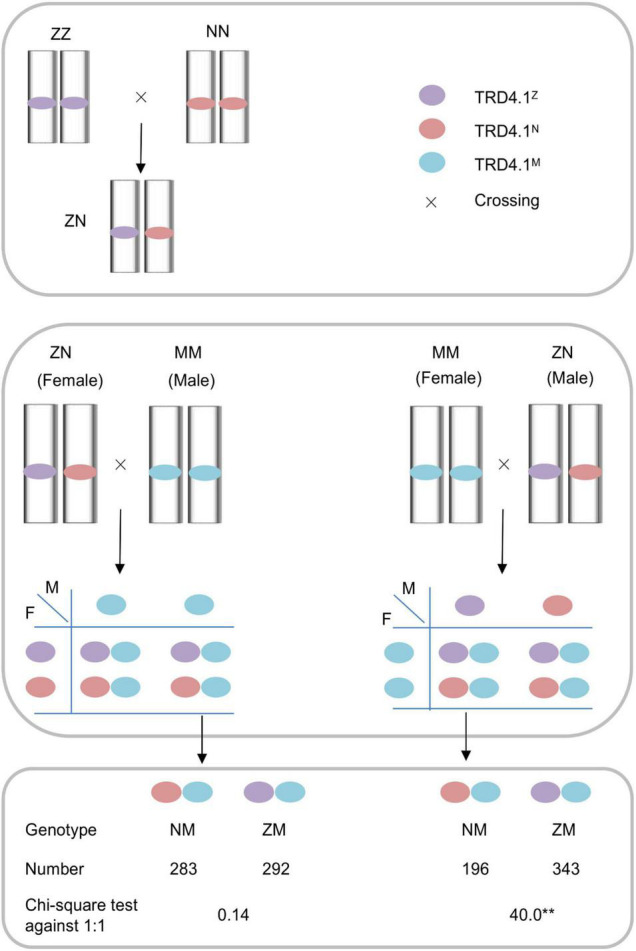
Schematic mode of gametic selection of *TRD4.1* in reciprocal crosses between the substitution lines ZN and MM within the Zhanshan97 background. ZN and MM contain heterozygous and homozygous alleles at *TRD4.1*, respectively. Three alleles N, Z, and M indicate Nipponbare, Zhanshan97, and Minghui63 alleles, respectively.

To track male- and female-specific transmission patterns for a given region using a reciprocal crossing test, diagnostic markers that were polymorphic among the parental lines (NIP, ZS97, and MH) were used for genotyping in the reciprocal cross progeny. The pattern of unequal frequencies through gametic selection is illustrated in Figure 1. If the F_1_ (ZN) is the male parent (or conversely the female), distortion of genotype (NM and ZM) frequencies in the progeny indicates the gametic selection at the F_1_-heterozygous locus.

### Pollen Fertility Observation

Pollen fertility was examined with the I_2_-KI staining method using mixed pollen grains of more than eight florets from the panicles of each plant, as previously described (Li et al., 2017). A microscope field with at least 200 pollen grains was observed. Pollen fertility was scored as the percentage of filled and stained grains in total grains (Kubo et al., 2011; Li et al., 2017).

## Results

### Detection of Transmission Ratio Distortion Regions on Chromosome 4 Using Chromosome Segment Substitution Lines-Derived Populations

To detect the TRD effect of the introduced NIP segments on chromosome 4, one of the CSSLs that contained the particular segments was crossed with ZS97 to produce a segregating population (name CSSL91-derived population). Graphical genotype analysis showed that CSSL91 carried four introduced NIP segments within the ZS97 background, two on chromosome 4 being targeted, and two on chromosome 2 (Supplementary Figure 1). Initially, the population (*n* = 191) was genotyped using four polymorphic markers, each located in the corresponding segment. The allele and genotype frequencies at the two markers (M1 and M2) on chromosome 2 showed normal-Mendelian segregation ratios. However, a non-Mendelian segregation of the two markers (M3 and M4) on chromosome 4 was observed in both allele and genotype frequencies (Table 1 and Supplementary Figure 1). Furthermore, eight additional consecutive markers (M5 to M12) distributed in the two TRD regions were all distorted significantly from the expected Mendelian ratios in the allele and genotype frequencies. The distortion of these markers was all biased toward ZS97 (Table 1 and Supplementary Table 2), suggesting that the ZS97 alleles at these two regions/loci (named *TRD4.1* and *TRD4.2*) were transmitted to the progeny at a higher frequency than the NIP alleles.

**TABLE 1:** Identification of *TRD4.1* and *TRD4.2* in the CSSL-derived segregating populations.

Loci	Marker	Position (Mb)	Genotype frequency					Allele frequency				Toward^a^
				
			P1	H	P2	χ^2^ (*df* = 2)	*P*-value	ZS97	NIP	χ^2^ (*df* = 1)	*P*-value	
*TRD4.1*	M5	6.46	71	95	20	28.05	8.1E-07	0.64	0.36	27.97	1.2E-07	ZS97
	M6	8.63	79	89	22	34.96	2.6E-08	0.65	0.35	34.20	5.0E-09	
	M3	11.65	73	95	22	27.37	1.1E-06	0.63	0.37	27.38	1.7E-07	
	M7	14.06	77	94	20	34.07	4.0E-08	0.65	0.35	34.02	5.5E-09	
	M8	14.65	73	97	19	30.90	1.9E-07	0.64	0.36	30.86	2.8E-08	
*TRD4.2*	M4	19.95	67	93	25	19.07	7.2E-05	0.61	0.39	19.07	1.3E-05	ZS97
	M9	20.09	74	94	23	27.30	1.2E-06	0.63	0.37	27.24	1.8E-07	
	M10	20.17	67	98	18	27.16	1.3E-06	0.63	0.37	26.24	3.0E-07	
	M11	20.20	70	85	26	22.10	1.6E-05	0.62	0.38	21.39	3.7E-06	
	M12	21.63	76	88	27	26.32	1.9E-06	0.63	0.37	25.14	5.3E-07	

*^a^The allele is preferentially transmitted in heterozygotes. P1, P2, and H indicate homozygous ZS97, Nipponbare (NIP), and heterozygous genotypes, respectively.*

### Validation of *Transmission Ratio Distortion 4.1* and *Transmission Ratio Distortion 4.2*

**FIGURE 2:**
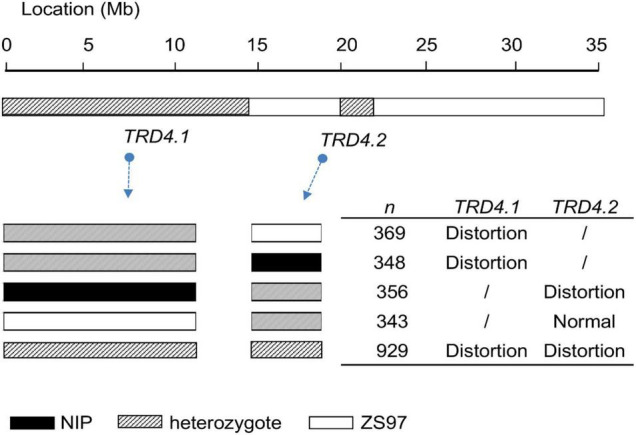
Detection of single and combined effects of *TRD4.1* and *TRD4.2* on transmission ratio distortion using five segregating populations (comprising *n* individuals), which were derived from corresponding chromosome segment substitution line that contains the particular heterozygous region (s), covering either *TRD4.1* or *TRD4.2* or both. The graphic genotype of the two loci in the relevant line is indicated.

To validate the effect of *TRD4.1*, two lines that carried heterozygous *TRD4.1* along with *TRD4.2* homozygous ZS97 or NIP were obtained and self-crossed to produce corresponding segregating populations. As a cluster of molecular markers showing TRD suggests that the chromosomal region may have one or more genes causing TRD, the representative marker (M7) was used to analyze the TRD effect at TRD4.1. A non-Mendelian segregation of *TRD4.1* was observed in these two populations (Figure 2 and Table 2). Moreover, the ZS97 alleles at *TRD4.1* were preferentially transmitted to the progeny in heterozygotes.

**TABLE 2:** Validation of *TRD4.1* and *TRD4.2* in four segregating populations.

Parental genotype	Representative Marker	Observed genotype frequency^a^						Observed allele frequency^b^				Toward^c^
			
		ZZ	H	NN	Sum	χ^2^ (*df* = 2)	*P*-value	ZS97	NIP	χ^2^ (*df* = 1)	*P*-value	
*TRD4.1* ^het^ */TRD4.2* ^ZS^	M7	128	178	63	369	23.4	8.5E-06	0.59	0.41	22.9	1.7E-06	ZS97
*TRD4.1* ^het^ */TRD4.2* ^NIP^	M7	122	174	52	348	28.2	7.7E-07	0.60	0.40	28.2	1.1E-07	ZS97
*TRD4.1* ^NIP^ */TRD4.2* ^het^	M12	136	181	39	356	53.0	3.2E-12	0.64	0.36	52.9	3.6E-13	ZS97
*TRD4.1* ^ZS^ */TRD4.2* ^het^	M12	81	184	78	343	1.9	3.9E-01	0.50	0.50	0.1	8.2E-01	Equal

*^a,b^Genotype and allele frequencies assayed by representative markers tightly linked with TRD4.1 or TRD4.2, respectively. ZZ, NN, and H indicate the homozygous ZS97 (ZS), Nipponbare (NIP), and heterozygous genotype (het), respectively. ^c^Toward means preferential allele transmission from heterozygotes. “Equal” indicates that both alleles are transmitted equally to the progeny.*

To check the effect of *TRD4.2*, two lines that harbored heterozygous *TRD4.2* and homozygous at *TRD4.1* either with ZS97 or NIP alleles were self-pollinated to produce segregating populations (Figure 2 and Table 2). Consistently, *TRD4.2* assayed by the representative marker M12 exhibited a significant TRD effect with the preferential transmission of the ZS97 alleles in the progeny of heterozygotes harboring homozygous *TRD4.1*^NIP^**. In contrast, normal segregation of *TRD4.2* was observed in the progeny of the heterozygotes when carried with homozygous *TRD4.1*^ZS97^ (Table 2).

An additional segregated population (*n* = 902) was also generated from the heterozygotes at both *TRD4.1* and *TRD4.2* (Figure 2). Frequencies of the nine genotypes assayed by two markers (M7 and M12) linked with *TRD4.1* and *TRD4.2* did not fit the Mendelian segregation ratio (χ^2^ = 185.5; *P* < 2.2 × 10^–16^), given the two loci are linked (Supplementary Table 2). In particular, the number of genotype homozygous *TRD4.1*^ZS97^*TRD4.2*^ZS97^** was more than that of *TRD4.1^NIP^*TRD4.2*^NIP^*, TRD4.1*^ZS97^*TRD4.2*^NIP^*, and *TRD4.1^NIP^*TRD4.2*^ZS97^* in the population. Collective data confirmed that TRD arose from both *TRD4.1* and *TRD4.2* and a preferential transmission of the ZS97 gametes at the two loci.

### Detection of Other Transmission Ratio Distortion Loci Conditions on *Transmission Ratio Distortion 4.1*

To determine whether *TRD4.1* affects other genomic regions on TRD, the BIL population that was previously developed from the cross of NIP and 9,311 (Zhang et al., 2020b) was divided into two subpopulations according to the *TRD4.1* genotype: SubN (*n* = 75) in which all lines had *TRD4.1*^NIP/NIP^** and SubJ (*n* = 325) that had *TRD4.1^9311/9311^* assayed by 10 consecutive bin-markers (from 13.47 to 15.87 Mb) at the TRD4.1 region (Supplementary Table 3). Four and ten regions were detected in SubN and SubJ, respectively. Among them, three TRD regions (*TR1.3*, *TR8.2*, and *TR12.1*) were common in both subpopulations. The other seven regions were identified only in SubJ. In particular, the *TRD4.2* region was detected only in SubN but not in SubJ (Supplementary Table 3). These results revealed the effect of *TRD4.1* on the detection of other TRD loci in the BIL population.

### Gametic Selection Leading to Transmission Ratio Distortion

To investigate whether *TRD4.1* and *TRD4.2* are involved in the gametic or zygotic factors, the population segregated at both TRD4.1 and TRD4.2 was analyzed. The results showed that the frequencies of heterozygous genotypes at either *TRD4.1* or *TRD4.2* were approximately 0.5. The observed frequencies of heterozygotes at either *TRD4.1* or *TRD4.2* exhibited no significant derivation from the expected frequencies (Supplementary Table 2). This non-distortion of heterozygote frequency may indicate a gametophytic barrier. In addition, normal pollen fertility (greater than 95%) of the nine genotypes revealed that TRD was not caused by pollen fertility (Supplementary Table 4). These results confirm that the gametic factors are involved in *TRD4.1* and *TRD4.2*.

To investigate whether the *TRD4.1* effect is caused by female or male gametic factors, a reciprocal test cross was made between NILs with a heterozygous (ZN) segment and a MM segment that contains homozygous Minghui 63 alleles at the locus (Figure 1). Segregation of the locus was assessed in a F_1_ progeny from the crosses. The progenies of the reciprocal crosses (NZ × MM and MM × ZN) were examined for TRD effects. The polymorphic markers M13 and M14 that were tightly linked with *TRD4.1* and which could distinguish the three genotypes (NIP, ZS97, and MH63) were used to classify two progeny genotypes (NM and ZM) of the crosses. The genotype frequency of *TRD4.1* showed normal-Mendelian segregation (no TRD) by the Chi-square test against the expected ratio of 1:1 (NM:ZM = 283:292; χ^2^ = 0.14; *P* > 0.70) in the cross of ZN × MM (*n* = 575), in which ZN was used as the female parent. The results suggest that NIP and ZS97 alleles were equally transmitted to the progeny. However, significant distortion of genotype frequencies (NM:ZM = 196:343; χ^2^ = 40.09; *P* < 2.5 × 10^–10^) was observed in the progeny of the cross of MM × NZ (*n* = 539), in which ZN was used as the male parent (Figure 1). In addition, normal pollen fertility (greater than 95%) of ZN was observed. These results indicate that the ZS97 allele was preferentially transmitted to the progeny over the NIP allele. Therefore, the distortion of genotype frequencies at *TRD4.1* in the progeny involved male gamete selection.

### Fine Mapping of *Transmission Ratio Distortion 4.1* and *Transmission Ratio Distortion 4.2*

**FIGURE 3:**
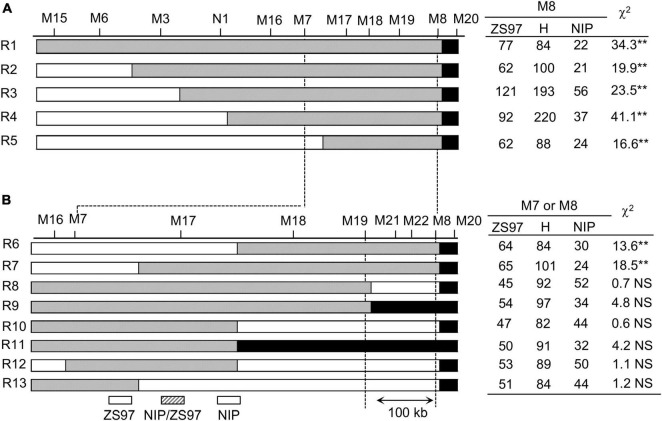
Fine-mapping of TRD4.1. R1 to R13 represents the recombinant individuals between M15 and M8. Their derived segregating populations were used to validate TRD of TRD4.1. **(A)** TRD analysis of five (R1 to R5) recombinant-derived segregating populations delimited TRD4.1 into a 590-kb interval. The polymorphic marker of M8 was used to investigate the genotypes of R1 to R5-derived populations. **(B)** Finely mapping of TRD4.1 to a 100-kb region between M8 and M19 using eight (R6 to R13) recombinant-derived populations. The marker M8 was used to investigate the genotypes of R6- and R7-derived populations. The marker M7 was used to investigate the genotypes of R8 to R13-derived populations. ZS97, H, and NIP denote ZS97, heterozygous, and NIP genotypes at TRD4.1, respectively. “**” Denotes significant distortion of the allele and genotype frequencies by chi-square test at *P* < 0.01. NS, no significance.

To narrow the region of *TRD4.1*, a mapping population comprising of approximately 6,500 individuals was developed from one plant that harbored only one heterozygous *TRD4.1* region and one homozygous *TRD4.2*^NIP^** within the ZS97 background. Initially, five recombinant individuals (R1 to R5) were obtained using several markers linked with *TRD4.1*. Then, the recombinants were self-crossed to generate five independent segregating populations for further genotyping (Figure 3). Based on frequencies of the genotypes in each recombinant-derived population using representative markers (M7 or M8), the distorted region covering *TRD4.1* was determined in the corresponding recombinant. *TRD4.1* was preliminarily mapped to an approximately 590-kb region between markers M7 and M8. For fine mapping of *TRD4.1*, eight recombinant individuals (R6 to R13) within the interval of M7-M8 were selected and generated eight independent recombinant-derived populations. TRD analysis based on frequency genotypes in recombinant-derived populations delimited *TRD4.1* into a 100-kb region between markers M19 and M8.

**FIGURE 4:**
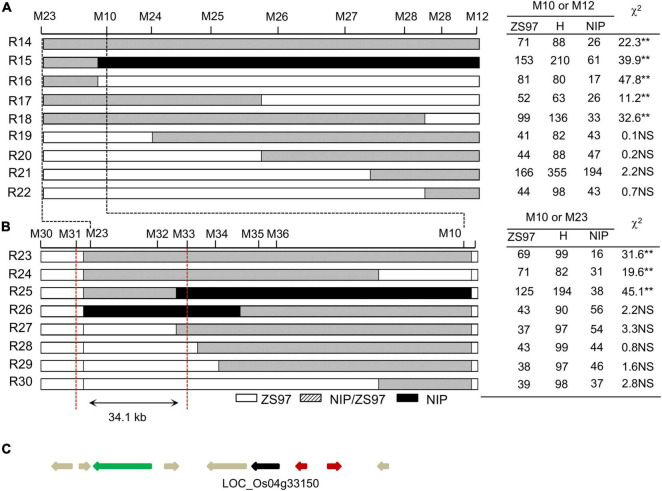
Fine-mapping and candidate gene analysis of *TRD4.2*. R14 to R30 represent the recombinant individuals between M23 and M12. **(A)** TRD analysis of recombinant-derived segregating populations delimited *TRD4.2* into a 600-kb interval. **(B)** Fine-mapping of *TRD4.2* to a 34.1-kb region between M31 and M34. ZS97, H, and NIP denote ZS97, heterozygous, and NIP genotypes at *TRD4.2*, respectively. “**” Denote significant distortion of the allele and genotype frequencies according to chi-square test at *P* < 0.01. NS, no significant distortion. **(C)** Annotated genes for *TRD4.2* based on the Nipponbare reference genome (http://rice.plantbiology.msu.edu/index.shtml), showing a candidate gene that is indicated by the dark arrow. The red, gray, and green arrows represent hypothetical proteins, expression protein, and transposon or retrotransposon, respectively.

Based on the same approach, fine-mapping of *TRD4.2* was conducted on a segregated population (composed of approximately 5,800 individuals) derived from a plant that carried only one heterozygous *TRD4.2* region and one homozygous *TRD4.1*^NIP^** segment in the ZS97 background. Seventeen recombinant individuals in the *TRD4.2* region flanked by markers M23 and M12 were obtained (Figures 4A,B). Based on TRD analysis of genotype frequencies in each of the 17 recombination-derived populations and genotyping with additional markers in the target region, *TRD4.2* was narrowed down to an approximately 34.1-kb region. This region contains nine predicted genes based on the reference genome^2^ (Figure 4C). Of them, LOC_Os04g33150 was the only one expressed gene, after removing those annotated as unknown, transposons/retrotransposons, or hypothetical proteins. It was specifically and highly expressed in the pre-emergence inflorescence, 5-d seed, and 25-d endosperm.^3^ This gene encodes a desiccation-related protein. Sequence differences that existed in the coding region between NIP and ZS97 may cause two amino acid changes.^4^ Therefore, LOC_Os04g33150 is the most likely candidate gene for *TRD4.2*.

## Discussion

The present study identified two linked loci *TRD4.1* and *TRD4.2*, and their epistatic interaction caused transmission ratio distortion in several CSSL-derived segregating populations, in which only a single or two NIP segments were targeted within the ZS97 background in rice. Both loci showed a preferential transmission of the ZS97 alleles to the progeny in heterozygotes. This severe TRD with a bias toward the *indica* allele is consistent with previous results in the NIP/9311 BIL population and other subspecific crosses (Wang et al., 2009; Reflinur et al., 2014; Zhang et al., 2020a).

One of the findings is that the TRD effect on *TRD4.2* was dependent on *TRD4.1* (Figure 1). *TRD4.2* showed severe TRD in the CSSL-derived populations in which the progeny harbored homozygous *TRD4.1*^NIP^**, but normal segregation in the progeny when it carried with homozygous *TRD4.1*^ZS97^ (Table 2). Furthermore, *TRD4.2* was detected only in SubN that had a fixed NIP genotype at the *TRD4.1* region in the NIP/9311 BIL population (Supplementary Table 3). However, *TRD4.1* displayed several allelic transmission biases no matter whether *TRD4.2* was homozygous or heterozygous NIP alleles (Figure 2). In addition, *TRD4.1* from the NIP decreased the incidence of TRD (63%) in a subset (SubN) of the BIL population. These results suggest that epistatic interaction plays an important determinant in the segregation patterns of TRD loci. This is the possible reason that some loci like *TRD4.2* have largely gone unnoticed in any previous study on rice.

Another finding in this study is that *TRD4.1* is possible only through male gametic selection among the progeny. TRD is a selection mechanism that could be caused by gametic and zygotic factors. In the present studies, using a series of CSSL- and NIL-derived populations, we found that the allele frequencies at *TRD4.1* and *TRD4.2* in the populations were significantly skewed toward ZS97 (Tables 1, 2); however, the heterozygous genotypes showed fertile pollen and normal segregation in the progeny (Supplementary Table 4). These results indicate that gametic selection was involved in *TRD4.1* and *TRD4.2*. Notably, we used a three-allele reciprocal crossing test to investigate whether there was a female or male gametic selection in *TRD4.1* and found that the severe transmission bias of *TRD4.1* was due to the male gametic selection of the NIP alleles. It is notable that *TRD4.1* was mapped in the common region where *SD4.1* was reported to affect segregation distortion and spikelet fertility in both inter- and intra-specific populations (Wan et al., 1996; Li et al., 2017; Zhang et al., 2020a). We further delimited *TRD4.1* into a 100-kb interval and *TRD4.2* into a 34.1-kb region with one candidate gene (Figures 3, 4), which would facilitate cloning the genes underlying the gametic factors for TRD. Further characterization and functional analysis of *TRD4.1* will be required to better understand the male gametic selection through gamete killer, gamete competition, and/or differential fertilization success.

## Conclusion

Two TRD regions (*TRD4.1* and *TRD4.2*) on chromosome 4 were identified and validated using CSSL-derived secondary populations. A significant digenic interaction between *TRD4.1* and *TRD4.2* affected TRD. Of them, *TRD4.2-*mediated TRD was dependent on the presence of *TRD4.1* alleles, but *TRD4.1* was not affected by *TRD4.2*. Moreover, *TRD4.1* and *TRD4.2* were delimited to approximately 100-kb and 34.1-kb intervals, respectively. Furthermore, we found that *TRD4.1* is male gametic in action with the preferential transmission of the *indica* ZS97 allele to the progeny. These findings would be helpful for cloning candidate genes and characterizing the molecular mechanisms underlying TRD.

## Data Availability Statement

The original contributions presented in the study are included in the article/Supplementary Material, further inquiries can be directed to the corresponding author/s.

## Author Contributions

SY designed and conceived the research. CZ, JW, XX, DW, and WS developed the populations. CZ, JW, XZ, XX, and ZY conducted the experiments. CZ and JW analyzed the data. CZ, JW, and SY wrote the manuscript. All authors read and approved the final manuscript.

## Conflict of Interest

The authors declare that the research was conducted in the absence of any commercial or financial relationships that could be construed as a potential conflict of interest.

## Publisher’s Note

All claims expressed in this article are solely those of the authors and do not necessarily represent those of their affiliated organizations, or those of the publisher, the editors and the reviewers. Any product that may be evaluated in this article, or claim that may be made by its manufacturer, is not guaranteed or endorsed by the publisher.

## Footnotes

1
https://www.r-project.org/

2
http://rice.plantbiology.msu.edu/

3
http://rice.plantbiology.msu.edu/expression.shtml

4
http://rice.hzau.edu.cn/rice_rs3/

## Supplementary Material

Supplementary Figure 1 Primarily mapping TRD regions on chromosome 4 using CSSL-derived populations. **(A)** Graphical genotype of CSSL91 showing four introduced Nipponbare segments encompassing TRD4.1 and TRD4.2 in the ZS97 background. **(B)** The allele and genotype frequencies at target regions (markers) showing non-Mendelian segregation by Chi-square test. ZZ, ZN, and NN represent ZS97, heterozygote, and NIP genotype, respectively.

Supplementary Table 1 Primers used in this study.

Supplementary Table 2 Nine genotypic frequencies at the loci TRD4.1 and TRD4.2 in a segregated population.

Supplementary Table 3 The genomic regions detected for transmission ratio distortion in two subpopulations fixed TRD4.1Nip or TRD4.19311.

Supplementary Table 4 Pollen fertility percentage of nine genotypes at TRD4.1 and TRD4.2.
