# Supplementary figures and images for: Fine Mapping of Two Interacting Loci for Transmission Ratio Distortion in Rice (Oryza sativa L.)
Source: Front Plant Sci. 2022 Mar 29;13:866276. doi: 10.3389/fpls.2022.866276 (PMC9002327; doi:10.3389/fpls.2022.866276)

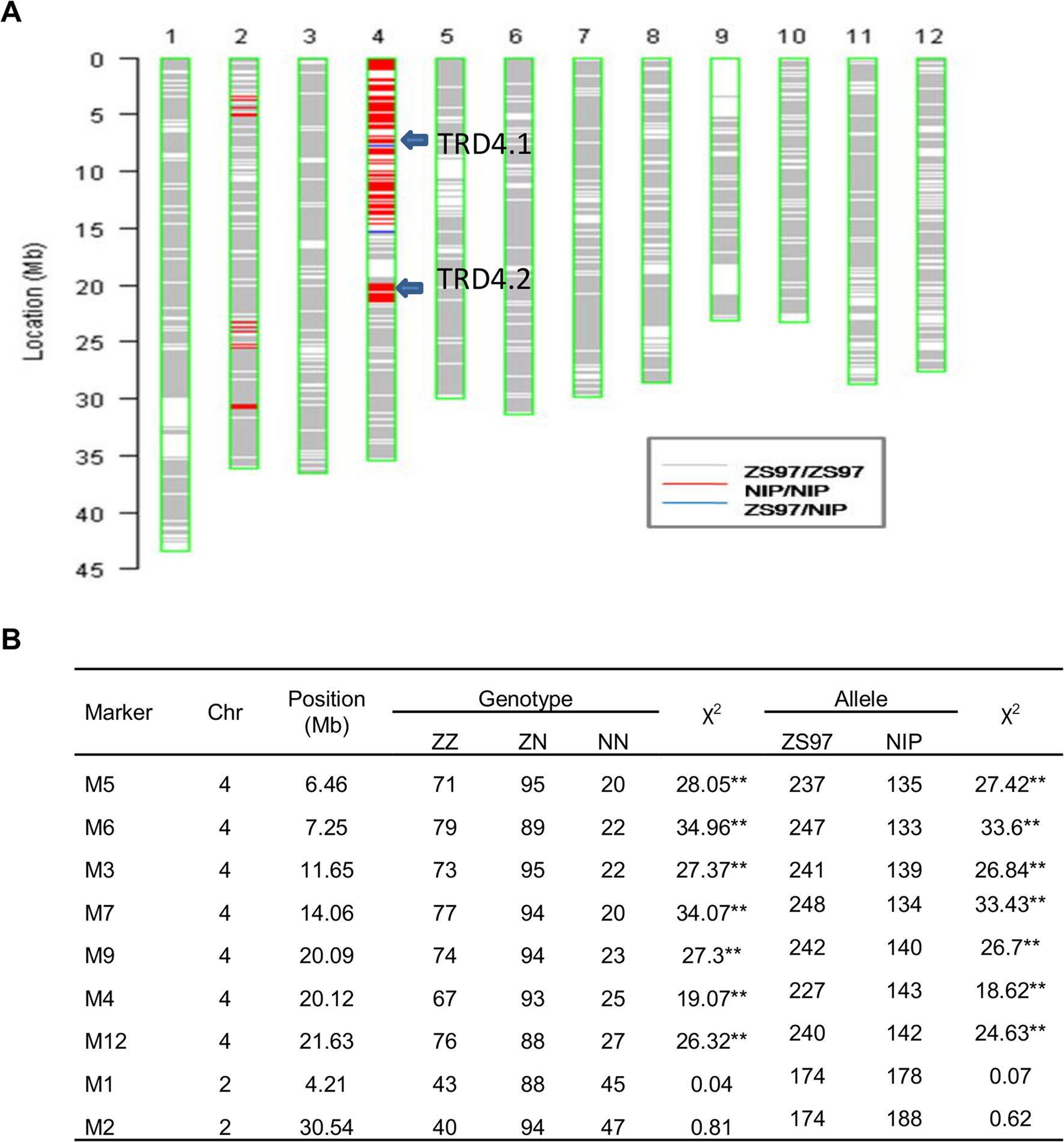

Supplement: Supplementary Figure 1 — Primarily mapping TRD regions on chromosome 4 using CSSL-derived populations. (A) Graphical genotype of CSSL91 showing four introduced Nipponbare segments encompassing TRD4.1 and TRD4.2 in the ZS97 background. (B) The allele and genotype frequencies at target regions (markers) showing non-Mendelian segregation by Chi-square test. ZZ, ZN, and NN represent ZS97, heterozygote, and NIP genotype, respectively. [file Image_1.TIF]
